# Natural Source, Chemical Classification and Medicinal Application of the Stilbene-Type Compounds: A Review of Structural Modification Around Stilbene Scaffold

**DOI:** 10.3390/molecules31132208

**Published:** 2026-06-23

**Authors:** Shengying Lin, Roy Wai-Lun Tang, Ran Duan, Ka Wing Leung, Tina Ting-Xia Dong, Karl Wah-Keung Tsim

**Affiliations:** Center for Chinese Medicine, Division of Life Science, The Hong Kong University of Science and Technology, Clear Water Bay, Kowloon, Hong Kong, China; lishlin@ust.hk (S.L.); roytwl@ust.hk (R.W.-L.T.);

**Keywords:** resveratrol, stilbene analogues, natural sources, chemical classification, structural modification, biological activity

## Abstract

Stilbene-type compounds are vital plant secondary metabolites that are classified under polyphenols and generally exhibit significant biological activities, as well as potential health benefits. These compounds, prevalent in food sources and medicinal plants, are recognized for their complex structures and their roles in plant defense mechanisms against environmental stressors. Despite their beneficial properties, the natural stilbenes face limitations related to their bioavailability and solubility, highlighting the need for chemical modifications to enhance their therapeutic efficacy. Studies have focused on structural modifications of the stilbene scaffold, including the introduction of carbon-based fragments, aiming to improve the compounds’ stability, selectivity, and overall biological activities. The development of stilbene analogues through chemical modifications not only expands the library of valuable stilbene-type compounds but also holds promise for new therapeutic applications in combating chronic diseases. This review summarizes current knowledge on the sources, biological activities, and chemical modifications of stilbene compounds, emphasizing their potential in healthcare and nutrition.

## 1. Introduction

Stilbenes are secondary metabolites synthesized by plants as a defense network against various external threats, including fungal or physical damage; ultraviolet radiation; viral or bacterial infections; and exposure to pesticides [1,2]. The family is characterized by a 14-carbon skeleton (C6–C2–C6) and consists of a number of stilbene family members. Stilbenes are recognized as phytoalexins—substances produced in response to infections and fall under the category of polyphenols. Their *Z*- and *E*-stereoisomers accumulate in the peels of vegetables during the plant’s developmental stages [1,2].

A diverse array of stilbene phytochemicals has been widely discovered, with structures ranging from monomers to octamers, each featuring different structural fragments at various positions, including glycosyl, hydroxyl, methyl, or isopropyl groups [3,4]. Some typical examples of stilbenes include resveratrol, viniferin, piceatannol, pterostilbene, and other derivatives. Out of these compounds, resveratrol, containing a 3,5,4′-trihydroxystilbene chemical scaffold and chemically belonging to the stilbene family, is particularly one of the most well-investigated phytochemicals for its broad spectrum of biological activities, such as anti-inflammatory responses and antioxidant effects, as well as its role in stimulating lipolysis in adipocytes [3,4].

The stilbene family, resveratrol in particular, has attracted significant scientific interest over the last two decades due to its diverse biological activities [5,6]. Research has demonstrated the capability of stilbenes in affecting redox balance, cell proliferation, mitochondrial function, angiogenesis, inflammation suppression, and adipocyte lipolysis. Recent investigations have explored other stilbene analogs, including monomers and dimers [5,6]. Despite the fact that some stilbenes have expressed their potential with significant biological effects, the efficacy of these phytochemicals can still be improved in order to achieve better levels for clinical applications [7,8]. Furthermore, there are also some issues that limit the application of stilbenes, such as low bioavailability and low stability. Therefore, there is still a large demand to conduct structural modifications around stilbene-type phytochemicals to further enhance their potencies, as well as pharmacological profiles [7,8]. This report serves as a review to not only summarize the natural sources, chemical classifications, and biological activities but also reveal recent structure-activity studies of the stilbene-type derivatives. Through keywords of “natural source of stilbenes”, “chemistry of stilbenes”, “biological activities of stilbenes”, “structural modification of stilbenes” in PubMed, CrossRef, and Google Scholar databases, we were able to obtain a series of previous research and review articles for our subsequent summary.

## 2. Sources and Chemical Classification

### 2.1. Natural Sources

Stilbenes are a significant group of plant secondary metabolites that fall under the category of polyphenols [9]. They are commonly found in various foods, such as grapes and peanuts, as well as in medicinal plants, e.g., *Hopea chinensis*, *Polygonum cuspidatum*, *Polygonum multiflorum*, *Vitis vinifera*, *Gnetum parvifolium*, and others [10]. Natural stilbenes are polyhydroxyphenolic compounds, created by replacing hydrogen atoms in different positions of benzene rings with hydroxyl groups, with stilbene scaffold serving as the main moiety. This group mainly includes the stilbene monomers, polymers, and heteromers [9,10].

Stilbenes are unevenly distributed across the plant kingdom. These compounds have been identified in over 275 species from more than 55 families, with a total of 1038 stilbenes being reported to date [9,10,11,12]. They are primarily found in 11 families, including Moraceae (MOR), Vitaceae (VIT), Leguminosae (LEG), Dipterocarpaceae (DIP), Gnetaceae (GNE), and so on, which together account for more than 84% of the overall occurrences, while fewer stilbenes have been reported in other families [11,12]. Notably, DIP and GNE families are particularly rich sources of natural oligomeric stilbenes [11,12].

The primary natural source of stilbenes is *V. vinifera*, part of the VIT family [13,14]. This species contains over 60 stilbenes, including monomers, like *trans*-resveratrol and piceatannol, as well as oligomers, which are typically found in their *trans* configuration. Stilbene derivatives are present in angiosperms, encompassing both monocots and eudicots [13,14]. These families showcase a wide range of botanical diversity, highlighting the extensive distribution of stilbenes across various plant groups, which make up more than 80% of the overall stilbene content. DIP and GNE families are crucial sources of naturally occurring oligomeric stilbenes, contributing over 50% of all reported natural oligostilbenes [15,16]. Conversely, the LEG family has the highest number of newly discovered monomeric stilbenes. Various oligomers, monomers, and unique stilbene hybrids have been isolated from multiple *Gnetum* species. Additionally, stilbene derivatives have been identified primarily in the genus *Welwitschia* and in the species of *Picea* [15,16]. Numerous studies have also found stilbenes, including *trans*-resveratrol and other stilbenoid compounds, in a variety of sources such as plant entomopathogenic bacteria, endophytic fungi, different sponges, mushrooms, and moth larvae [15,16].

Resveratrol, a typical stilbene analog, has been obtained from various sources, including grapes, peanuts, bilberries, and cranberries, from more than 34 families of foods, plants, and other natural sources [17,18]. The major source of dietary stilbenes, especially *cis*-resveratrol, is the *Vitis* genus of the VIT family, particularly found in grapes and red wine. While stilbenes are predominantly present in grapes and wine, their concentrations can vary significantly [17,18]. The key factors, such as soil type, grape variety, temperature, and pathogen infection, are likely to have an impact on stilbene production. As part of the plant’s natural defenses, the levels of stilbenes could fluctuate due to the environmental conditions. Furthermore, the processing of grapes into juices or wines is able to alter stilbene content because of the winemaking practices. The glucosylated form of resveratrol, known as piceid, can also be identified and varied in multiple vines and *Vitis* species [19,20]. Other types of stilbenes, such as resveratrol, piceatannol, and pterostilbene, as well as dimeric forms, e.g., pallidol, ε-viniferin, and δ-viniferin, have been discovered and recognized. Additionally, the homodimers of resveratrol–resveratrol and piceatannol–piceatannol, along with their heterodimers and chemically modified dimers (such as O-glycosylated, methoxylated, and oxidized forms), as well as multiple trimers and tetramers, have been discovered and determined, highlighting the chemical diversity of stilbene derivatives derived from plants and wines [19,20].

### 2.2. Chemical Classification

#### 2.2.1. Resveratrol

**Figure 1:**
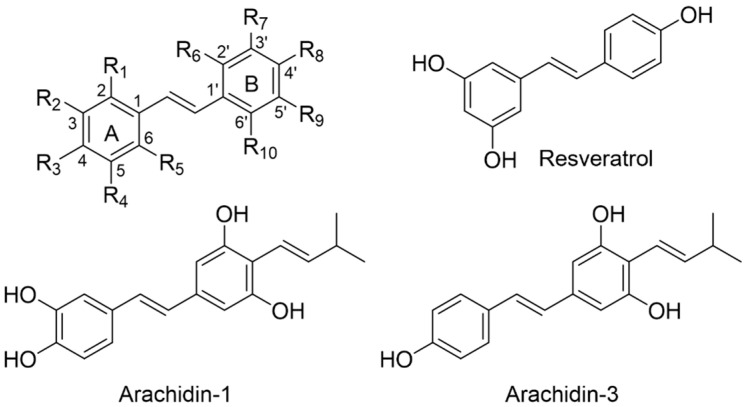
Chemical skeleton of the stilbene family and examples of prenylated stilbenes. Chemical structures were obtained from PubChem database (https://pubchem.ncbi.nlm.nih.gov/, accessed on 1 February 2026) and ChemDraw software (https://revvitysignals.com/products/research/chemdraw, version 20.0, accessed on 1 February 2026).

Stilbenes, belonging to the non-flavonoid polyphenolic class, constitute a typical structure featuring a 14-carbon skeleton (C6–C2–C6) composed of two aromatic benzene rings linked through an ethylene bridge (Figure 1) [21,22]. In general, one of the aromatic rings contains two hydroxyl groups, while another ring is likely to have both hydroxy and methoxy groups located at different positions [21,22]. The ethylenic scaffold allows for the existence of two stereoisomers: *trans*-stilbene (*E*-stilbene) and *cis*-stilbene (*Z*-stilbene), of which *E*-stilbene is usually more stable and abundant in nature [22,23,24]. As one of the most crucial analogs from the stilbene family, resveratrol (3,5,4′-trihydroxystilbene) generally exists in two forms, *cis*-(*Z*) and *trans*-(*E*), and the *trans*-form could readily go through isomerization to the *cis*-form under heat or ultraviolet irradiation conditions [22,23,24].

#### 2.2.2. Prenylated Stilbene Derivatives

Prenylated stilbene analogs contain extra C-C bonds in their core scaffold, with additional terpenic substituents, such as prenyl, geranyl, and hydroxyprenyl groups [25,26]. The majority of functional groups originated from isoprenoid groups, which usually constitute 5–10 carbon atoms and are transformed via kinetic reactions in various fungi, plants, and microorganisms. The most favorable positions allowing for terpenic substitutions are at C-2, C-4, C-6, and/or C-5′ of the central moiety [25,26]. Modifications, including cyclization and hydroxylation, can also take place, resulting in diverse analogues that exhibit improved bioavailability and lipophilicity. These types of stilbenes are mainly identified in specific plant families, such as MOR and LEG, with over 400 derivatives identified from natural sources [25,26,27].

The prenylation of the stilbene backbone is a key factor that leads to the diversity of these secondary metabolites found in plants [28,29,30]. Such a modification enhances the lipophilicity and membrane permeability of stilbenes and also has a positive impact on their bioactivities. In in vitro studies, prenylated stilbenes have demonstrated equivalent or improved bioactivities as compared to their non-prenylated derivatives, such as resveratrol [28]. For instance, the prenylated stilbenes, arachidin-1 and arachidin-3 (Figure 1), exhibited improved metabolic profiles in comparison to their non-prenylated analogues, including piceatannol and resveratrol [29,30].

#### 2.2.3. 2-Phenyl-Benzofuran Analogues

**Figure 2:**
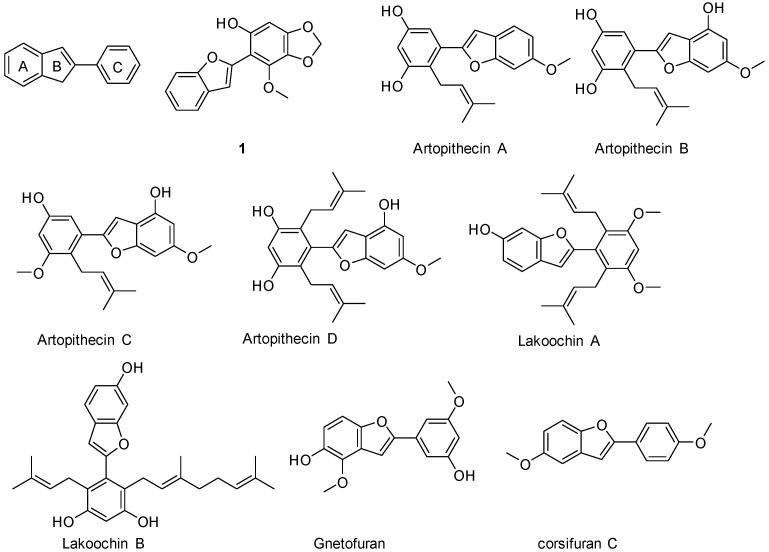
Chemical skeleton and examples of 2-Phenyl-Benzofuran analogs. Chemical structures were obtained from PubChem database (https://pubchem.ncbi.nlm.nih.gov/, accessed on 1 February 2026) and ChemDraw software (https://revvitysignals.com/products/research/chemdraw, version 20.0, accessed on 1 February 2026).

The 2-phenyl-benzofuran derivatives represent another category of stilbene-type phytochemicals, typically being characterized by a benzofuran core moiety (rings A–C) and a phenyl fragment (ring B), located at the C-2 position (Figure 2) [31,32]. This structure is commonly referred to as 2-phenyl-benzofuran, although some studies call it “2-arylbenzofuran” or “aryl benzofuran.” However, the latter two terms can be misleading, as “aryl” can be indicated as any heterocycle, not necessarily as the phenyl ring [31,32]. Despite being recognized for over 60 years, naturally existing 2-phenyl-benzofuran remains relatively obscure within the stilbene family. In 1958, a novel compound was identified in yeast and then was recognized as 2-(6-hydroxy-2-methoxy-3,4-methylenedioxyphenyl)-benzofuran (**1**, Figure 2). Thereafter, more than sixty additional 2-phenylbenzofuran derivatives have been successfully isolated from various plants [31,32,33].

This group of arylbenzofuran derivatives represents a unique type of stilbene characterized by a C7-O-C7 linkage, and they are phytochemical compounds with various pharmacological properties [31,34]. As an example, 2-aryl benzofurans were identified from only a limited number of known plant categories, including MOR and VIT [31,34]. Furthermore, artopithecins A-D are prenylated derivatives derived from *Artocarpus pithecogallus*, and lakoochin A contains two prenyl groups (Figure 2), while lakoochin B features a prenyl group at the C-2 position and a geranyl fragment at C-6 from the *Artocarpus lakoocha* species [31,32]. On the other hand, gnetofuran was first extracted and identified from a *Gnetum* source, while corsifuran C was discovered from *Corsinia coriandrina* [31,32].

#### 2.2.4. Carbon-Substituted Derivatives

**Figure 3:**
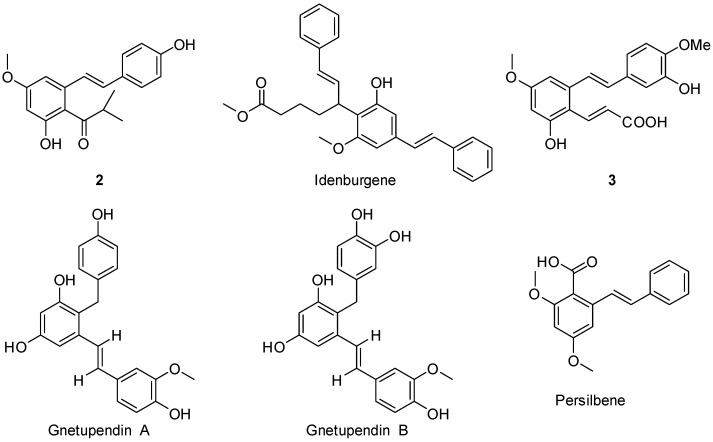
Chemical examples of Carbon-Substituted derivatives. Chemical structures were obtained from PubChem database (https://pubchem.ncbi.nlm.nih.gov/, accessed on 1 February 2026) and ChemDraw software (https://revvitysignals.com/products/research/chemdraw, version 20.0, accessed on 1 February 2026).

Carbon-substituted stilbenes, commonly referred to as carbo-stilbenes, are usually molecular structures in which the carbon skeleton of the stilbene scaffold is expanded by the introduction of carbon-based fragments, including phenyl and alkyl groups [35,36]. Such modifications generally change the chemical and physical properties of the parental molecule, affecting its reactivity as well as solubility. The structural alteration is employed to investigate various properties and to develop analogues with various biological activities, such as anti-inflammatory, anti-cancer, and other biological effectiveness [35,36,37]. They consist of a central ethylene scaffold with phenyl groups, along with additional C-C bonds that integrate more carbon atoms into the core moiety. For example, the geranyl groups, containing 5–10 carbon units, respectively, are usually incorporated into the core structure to generate carbon-substituted analogs [36,37]. Isobutanoyl derivatives, such as 1-{2-hydroxy-6-[(lE)-2-(4-hydroxyphenyl) ethenyl]-4-methoxyphenyl}-2-methyl-1-propanone (**2**, Figure 3) and its 2,4-dihydroxy derivative, have been discovered from *Ekebergia benguelensis*. Idenburgene, obtained from the *Cryptocarya idenburgensis* species, while gnetupendins A and gnetupendins B, sourced from *Gnetum pendulum*, contain a benzyl fragment. Other examples include 2-carboxy stilbene (known as persilbene) from *Polygonum persicaria*, analogue **3** (Figure 3) from *Convolvulus hystrix*, and a 5-O-terpinen-4-yl derivative obtained from *Alpinia katsumadai*, all of which are recognized as carbon-substituted derivatives of resveratrol [35,37]. It is worth mentioning that the existence of these additional carbon groups can lead to poor solubility in some cases, with a small number of carbo-stilbenes appearing as black solids [35].

## 3. Biological Activities of Stilbene-Type Derivatives

### 3.1. Antioxidant

The accumulation of free radicals in the body and the resulting oxidation usually lead to an imbalance in antioxidant reactions and the subsequent oxidative stress [38,39]. The majority of free radicals in cells, primarily generated by mitochondria, are generally produced by reactive oxygen species (ROS). The excess of ROS disrupts metabolic functions, damages cells and tissues, and contributes to various health issues [38,39]. Antioxidants are complexes that help neutralize and capture free radicals, thereby mitigating the damage inflicted on the body by these free radicals [38,39].

**Figure 4:**
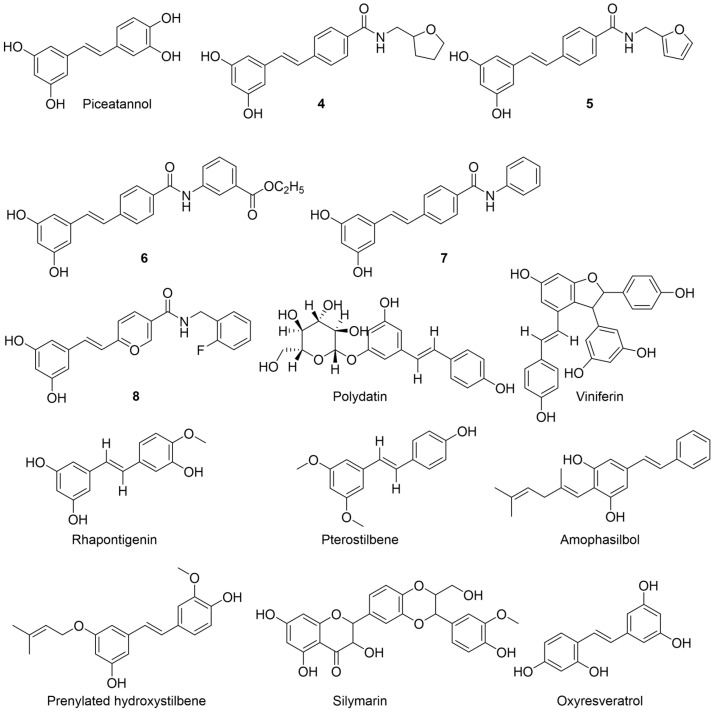
Biological activities of stilbene analogues. Chemical structures were obtained from PubChem database (https://pubchem.ncbi.nlm.nih.gov/, accessed on 1 February 2026) and ChemDraw software (https://revvitysignals.com/products/research/chemdraw, version 20.0, accessed on 1 February 2026).

Naturally occurring stilbene analogues have been widely reported to express fundamental antioxidant activities in the fields of food and medicine [40,41]. Fauconneau et al. [41] evaluated the antioxidant effectiveness of flavonoids (such as anthocyanins and catechins) and non-flavonoids (e.g., stilbenes) based on their ability to inhibit Fe^2+^-mediated lipid peroxidation in microsomes, as well as their effects on Cu^2+^-regulated lipid peroxidation in low-density lipoproteins. The findings indicated that both the quantity and positioning of hydroxyl groups have made significant contributions to the antioxidant activity of stilbenes. From a chemical perspective, the hydroxy group of stilbene derivatives plays a crucial role in antioxidant activity, primarily due to its ability to stabilize through resonance in the conjugating double-bond skeleton [42,43]. For instance, resveratrol demonstrated good antioxidant properties during tumor progression, interfering with its advancement by inhibiting multiple phases of the cell cycle. Piceatannol (Figure 4) is a stilbene-type phytochemical commonly found in berries and displays better antioxidant activity than resveratrol (1.53 µM vs. 63.2 µM) in B16F10 melanoma cells, according to Piotrowska et al. [44].

Caruso et al. [45] have examined resveratrol and its derivatives using ab initio calculations and crystal structure analysis, concluding that the *para*-4′-hydroxy group is more acidic in comparison with the other two *meta*-hydroxy groups, and H-atom movement is the primary mode of action by which stilbene analogues typically scavenge free radicals (Table 1). To compare the antioxidant activities of *cis*- and *trans*-resveratrol stereoisomers, Orallo and co-workers investigated the potential in vitro effects of the two isomers on production of ROS and reactive nitrogen species (RNS) during the respiratory burst of rat peritoneal macrophages. The findings indicate that the *cis* isoform is less effective than the *trans* isoform [46]. Stivala et al. [47] assessed the antioxidant effects of six resveratrol derivatives through in vitro assay by measuring their ability to attenuate citronellal thermo-oxidation or their radical scavenging capacity by using the DPPH free radical. As a result, it was concluded that reducing the stilbene double bond to a single bond could result in dihydroresveratrol that significantly diminishes the antioxidant capacity as compared to resveratrol, which was in line with the hypothesis above [47].

Jung et al. [48] conducted intensive structure-activity-relationship studies and obtained a group of stilbene analogues for the following in vitro evaluation. It was observed that compound **4** (Figure 4), containing an N-tetrahydrofuran-2-ylmethyaminocarbonyl fragment at the 4′-position on the phenyl ring, exhibited lower activity compared to compound **5,** having an N-furan-2-ylmethylaminocarbonyl group in the same position [48]. Furthermore, compound **6**, featuring an N-(3-ethoxycarbonylphenyl) aminocarbonyl group, demonstrated more robust antioxidant activity than the compound **7** lacking substitution at that position. The findings imply that an acyclic amine group expresses more significant antioxidant effectiveness than a cyclic amine group at the 4′-position of the phenolic ring (e.g., the compound **8**) [48]. Additionally, the introduction of an aromatic ring decreases potency as compared to an aliphatic ring (as seen in compound **7**) [48].

### 3.2. Anti-Inflammatory

The inflammatory response is a natural immune reaction that exists in the body when exposed to harmful substances [80,81]. Such a response can be induced by various factors, including the invasion of pathogens, somatic damage to host cells, and exposure to harmful toxins. Both inflammation and oxidative stress play significant roles in the development of numerous diseases [80,81]. ROS is crucial in initiating the expression of Toll-like receptors (TLRs) 2 and 4, and this could subsequently lead to the acceleration of inflammatory response. Besides, ROS can enhance the production of pro-inflammatory cytokines, which further results in promoting an inflammatory response [80,81]. Inflammation inducers are generally categorized into two classes, i.e., damage-associated molecular patterns (DAMPs), which result from host cell or tissue damage, and pathogen-associated molecular patterns (PAMPs), arising from the entry of pathogens and their cell wall components that activate the immune system [80,81].

Resveratrol was shown to epigenetically mediate cell growth and apoptosis while enhancing the expressions of anti-inflammatory cytokines IL-4 and IL-10, as well as miR-Let7a, with an IC_50_ value of 19 µM [49]. Furthermore, it decreases the levels of TNFα and IL-6. Similar effects have also been observed with *cis*- and *trans*-gnetin H RSV derivatives [49]. Polydatin acts as a Sirt-1 initiator and, in combination with resveratrol, can slow down the activation of ICAM1 and TNFα in a clinical trial at a dosage of 25.0 mg/kg/day [50]. Other stilbenes, including piceatannol and viniferin, are able to attenuate the LPS-triggered expressions of TNFα and IL-1β in RAW 264.7 cells [51]. As reported, the production of TNFα, triggered by LPS, was inhibited by piceatannol in comparison with resveratrol, and it was also found to inhibit T-cell receptor signaling in the murine splenocytes at relatively low concentrations (1–10 µM) [51].

In parallel, stilbenes have been reported to mitigate inflammation-related allergies. Piceatannol (Figure 4) blocks the allergic inflammatory response in mast cells by mediating MAPK phosphorylation [52]. Rhapontigenin was shown to significantly suppress histamine production from mast cells, reduce hyaluronidase (HYAL) expression and diminish passive cutaneous anaphylaxis reactions with an IC_50_ value of 0.25 µM [53]. An analogue of rhapontigenin, desoxyrhapontigenin, exhibits anti-inflammatory potency through mediating downstream signaling pathways and attenuates the NF-κB and MAPK pathways in macrophages [82].

### 3.3. Anti-Cancer

Cancer prevention has been one of the most extensively investigated research fields. Given the multiple mechanisms involved in driving towards cancerous disease, diverse medicinal approaches are required to serve as clinical options [83,84]. To date, natural phytochemicals featuring a stilbene scaffold have shown robust potential in cancer prevention by targeting various cellular signaling pathways [83]. Stilbene-type analogues generally inhibit the metabolic activation of pro-carcinogens through suppressing different isoforms of cytochrome P450 enzymes, leading to preventing the activation of carcinogenesis in tumor cells [84].

As a key member of the stilbene family, resveratrol offers protection from cancer through triggering phase II metabolism of carcinogens and induces the elimination of carcinogens from our bodies (Table 1) [54,55]. For instance, resveratrol has been shown to increase the performance of metabolizing enzymes, including uridine-5-diphospho (UDP) glucuronyl-transferase and NADPH/quinone oxidoreductase in mouse epidermis at a dosage of 80 mg/kg/day [54]. Additionally, resveratrol may inhibit cancer progression by blocking the activities of transcriptional and growth factors, such as ATF3, p53, and FoxO, and such a pathway could play a crucial role in cancer activation and promotion in cell models [55]. Additionally, Carter et al. [56] recently revealed that resveratrol has been found to prevent cell proliferation and trigger cell apoptosis in different cancerous animal models, such as liver, prostate, breast, colorectal, and pancreatic cancers, at dosages of 10–100 mg/kg/day.

Stilbene-type phytochemicals have been widely reported to exhibit a broad spectrum of biological activities. Pterostilbene, for example, demonstrates good inhibitory effects on a colon cancer cell model owing to its decent lipophilicity [57]. Besides, pterostilbene was also shown to promote the expression of tumor-suppressive microRNAs, as well as argonaute 2 (Ago2), a key component of RNA interference in human breast cancer cells [57]. Piceatannol, a derivative of resveratrol, has been found to inhibit a wide range of cancer types. It inhibits the proliferation and growth of AH109A hepatoma cells through mechanisms such as cell cycle arrest, antioxidant, and apoptosis effects with an IC_50_ value of 31.6 µM [58]. Moreover, two independent studies have implied that piceatannol reduced the metastasis of prostate and breast cancer cells by inhibiting matrix metallopeptidase (MMP)-9 [58,59]. Recently, a group of newly identified C- and O-prenylated piceatannols was discovered in propolis from Kangaroo Island in South Australia. For instance, prenylated hydroxystilbene (Figure 4) could inhibit cell growth and have antiproliferative effects against the leukemia K562 cell line at relatively low concentrations (5–30 µM) [59].

### 3.4. Cardiovascular Disease

Cardiovascular diseases (CVDs), also known as heart diseases, are leading causes of mortality worldwide. The World Health Organization (WHO) has reported that approximately 17.9 million people die annually due to CVDs, such as coronary heart disease (CHD) and cerebrovascular [85]. Recently, stilbene-type analogues have gained significant attention for their protective effects against CVDs. These compounds feature aromatic phenolic rings that can lose an electron, resulting in the formation of hydrogen free radicals [62]. The stilbene family could act as reducing agents, as well as free radical scavengers, and subsequently contribute to a potential mode of action that leads to cardioprotection [62].

Several lines of evidence have identified various target sites and mechanisms through which stilbenes exert beneficial effects against CVDs (Table 1) [60,61,62]. In 2012, Tomé-Carneiro and colleagues conducted a parallel, randomized, triple-blinded investigation that revealed a reduction in C-reactive protein, TNF-α, PAI-1, and the IL-6/IL-10 ratio, along with an increase in the expression of the anti-inflammatory cytokine IL-10 with an administration of resveratrol at 8 mg/day as compared to the blank and resveratrol-deficient groups [60]. This suggested that resveratrol consumption improved the fibrinolytic status of patients and reduced the occurrence of CVDs. Another investigation, conducted by Wong et al. [61], analyzed the effects of resveratrol supplementation on vascular activities and flow-mediated dilation in a double-blind study. They found that six weeks of resveratrol intake was well tolerated and led to a 23% increase in flow-mediated dilation, as compared to the placebo group. Additionally, a single dose of 75 mg of resveratrol supplementation per day enhanced the flow-mediated dilation response by 35% in comparison with the placebo, suggesting a significant anti-CVD effect [61].

Other stilbene compounds also demonstrate decent potency against CVDs. Silymarin (Figure 4), derived from milk thistle seeds, has been shown to improve CVD-associated risk factors, including LDL-C and total cholesterol (TC) levels [62]. Derosa et al. [63] studied subjects with dyslipidemia and revealed that silymarin, in combination with berberine, significantly attenuated the levels of LDL-C and TC after the treatment with a dosage of 105 mg twice a day for 3 months, as compared with placebo. To date, there have been limited studies regarding the effectiveness of pterostilbene in regulating blood pressure. A randomized and placebo-controlled clinical study revealed that a high dose of pterostilbene (approximately 125 mg twice daily) decreased blood pressure (both systolic and diastolic), while a relatively low dose (approximately 50 mg twice daily) failed to achieve similar results [64].

### 3.5. Neuroprotective Effect

The neuroprotective effects of the stilbene family primarily stem from their promising antioxidant and anti-inflammatory properties. Neurodegenerative diseases, such as Parkinson’s and Alzheimer’s diseases (AD), are linked to oxidative stress and mitochondrial dysfunction, which further result in neuronal loss and impaired function [86,87,88]. AD is a progressive neurodegenerative condition affecting the cortex and hippocampus, eventually resulting in cognitive decline. Although its precise pathology is unclear, the presence of β-amyloid peptides in the areas of learning and memory has been identified as a hallmark of AD [86,87]. The accumulation of amyloid-β is crucial in driving the pathogenesis of AD, and it has been shown to cause oxidative damage in neurons by triggering DNA oxidation, lipid peroxidation, and protein oxidation [87,88].

The neuroprotective effect of resveratrol has been illustrated, demonstrating its efficacy in alleviating the activities of aging and neurodegenerative disorders, including Parkinson’s and Alzheimer’s diseases [65,66]. In a cell model related to Parkinson’s disease, resveratrol has displayed potential as an effective agent at a concentration of 25 µM through reducing cytotoxicity mediated by MPP+. Such efficacy operates via the AKT/GSK-3β signaling pathway, emphasizing resveratrol’s capacity to counteract one of the main pathologies in Parkinson’s disease and to reduce neuronal damage [65,66].

Pterostilbene has also been found to exert neuroprotective effects and target high glucose-induced injury in neuroblastoma cells (Table 1). In this context, pterostilbene reduces cell death and production of ROS at relatively low concentrations (2.5–10 µM) [67,68]. Furthermore, it enhances the performance of mitochondrial complexes I and III, increases mitochondrial cytochrome C levels, and improves mitochondrial membrane potential. In addition, the treatment with pterostilbene could elevate the levels of Nrf2, HO-1, and glutathione S-transferase (GST), suggesting the crucial role of pterostilbene in reducing neuronal oxidative stress [67]. Similarly, pterostilbene could also mitigate the glutamate-induced oxidative stress in neurons through the Nrf2 signaling pathway [68].

In another study, Andrabi and co-workers reported the neuroprotective activity of oxyresveratrol through a murine middle cerebral artery occlusion (MCAO) in vivo model [69]. Oxyresveratrol was found to inhibit brain infarct volume in MCAO rats at dosages of 10 or 20 mg/kg, reduce neurological deficits due to ischemia/reperfusion injury, block the production of cytochrome C, and inhibit the initiation of caspase-3, which suggested that oxyresveratrol contains decent neuroprotective effects [69,70].

### 3.6. Obesity

Obesity is defined by an excess of adipose tissue resulting from increased caloric intake and/or insufficient energy expenditure [89]. Specifically, a body mass index (BMI) greater than 30 and a waist circumference exceeding 88 cm for women and 94 cm for men are significant risk factors for developing cardiovascular disease (CVD). SIRT1 is a molecular target of resveratrol and plays a vital role in maintaining mitochondrial homeostasis and regulating cellular energy metabolism [89,90]. Resveratrol notably increases the level of SIRT1 protein, thereby influencing cellular energy consumption. Furthermore, resveratrol has a positive impact on the AMPK pathway, which is essential for controlling lipogenesis in adipose tissue [91]. Studies involving resveratrol as supplementation in mouse models have shown some effectiveness in reducing fat regeneration in muscle, improving AMPK activity, and enhancing mitochondrial biogenesis (Table 1). Together, these results indicate that resveratrol is able to support muscle regeneration in obese individuals to some extent [91].

Resveratrol-rich wines are recognized as an excellent source of *trans*-resveratrol and its derivatives, such as ε-viniferin, a dimer of resveratrol that demonstrates a superior effect against adipogenesis in metabolic disorders [71]. Besides, ε-viniferin has been shown to be more effective than *trans*-resveratrol because of its significant anti-obesity and anti-inflammatory properties, with an IC_50_ value of 33.5 ± 9.7 µM in animal models being subjected to a high-fat diet [71]. Other research also emphasized that resveratrol, along with its dimers ε-viniferin and δ-viniferin, could contribute to the prevention of atherosclerosis through related molecular mechanisms [71,72].

Like resveratrol, pterostilbene possesses decent effects against obesity. Pterostilbene was found to inhibit fat accumulation within adipose tissues through blocking lipogenesis and promoting oxidation of fatty acid in the liver [73,74]. Pterostilbene has been shown to enhance AMPK and acetyl-CoA carboxylase performance within adipose tissue. In a Zucker fa/fa rat model, pterostilbene enhanced thermogenesis within brown adipose tissue at dosages of 15–30 mg/kg by upregulating the activities of uncoupling protein 1 (UCP-1), a vital regulator in the thermogenesis process [73,74].

### 3.7. Other Biological Effects

Diabetes is a chronic metabolic disorder linked to inflammatory and oxidative stress. Owing to its anti-inflammatory and antioxidant properties, resveratrol is able to slow down the progression of diabetic complications [92,93]. Previous studies in the C57BL/6J mice model have suggested that resveratrol may contain anti-diabetic properties by improving insulin resistance, enhancing impaired insulin signaling pathways, and reducing pancreatic beta cell apoptosis as well as cell dysfunction at dosages of 8–16 mg/kg/day (Table 1) [75]. In parallel, another stilbene analogue, pterostilbene, has been shown to enhance glycemic control in insulin-resistant obese rats by upregulating hepatic glucokinase activity and improving glucose uptake in skeletal muscle in a male Wistar rat model at 10–40 mg/kg/day [76]. Another in vitro study demonstrated that pterostilbene showed a protective effect on pancreatic beta cells from oxidative stress and cell apoptosis [77].

Melanin, a pigment produced by melanocytes during melanogenesis, serves to protect the skin from the harmful effects of ultraviolet (UV) radiation [94]. However, excessive melanin production in certain acquired hyperpigmentation diseases can lead to skin issues. Additionally, the dysregulation of melanogenesis has been linked to more aggressive melanotic melanomas, possibly due to increased expression of cytotoxic and immunosuppressive intermediates, including quinones, semiquinones, and ROS [94,95]. Resveratrol was found to control the UVB-mediated hyperpigmentation in the skin tissue of guinea pigs at a dosage of 1% solution (200 μL)/day [79]. On the other hand, resveratrol could attenuate melanogenesis-related proteins, including TRYP1, TRYP2, and MITF within melanoma cells [79]. Another stilbene derivative, piceatannol, was believed to contain an anti-melanogenetic effect, as it significantly reduced melanin content and exhibited stronger anti-tyrosinase activity than both kojic acid and resveratrol through B16F10 cell models at concentrations from 5 to 50 µM [78].

## 4. Structural Modification of Stilbenes

Stilbene-type analogues are a group of versatile compounds composed of two aromatic rings being connected by an ethylene bridge [96]. For decades, the stilbene scaffold has been recognized for its fundamental biological effects. Plants produce natural stilbenes to defend against stressors, including heat exposure, UV radiation, and fungal or bacterial infections [18,97]. Nevertheless, the biological efficacies of stilbenes have remained relatively low, as well as bioavailability and chemical stability, which together limit the development of stilbenes for the subsequent pharmacological process and clinical applications. Therefore, there is still a large requirement to carry out intensive structure-activity-relationship studies around the stilbene scaffold with an aim to further improve its potency and bioavailability [18,97]. This section serves as a review to summarize the reported structural modification around the stilbene scaffold.

### 4.1. Modification Around the Aromatic Ring

The strategic modification of the stilbene scaffold, particularly at both aromatic rings (Figure 1), represents a key avenue in enhancing its diverse bioactivities. This specific location is often targeted for the introduction of various functional groups and larger structural motifs, such as curcumin, chalcone, or nitrovinyl moieties. These additions are not random; they are designed to leverage the synergistic interactions and inherent properties of the introduced structures with the stilbene core [98,99]. The impact of these modifications could be profound, leading to significant improvements across a spectrum of therapeutic applications. Enhanced anti-inflammatory and anti-cancer properties are commonly observed, suggesting that these structural alterations can modulate key cellular signaling pathways involved in disease progression [98,99]. Furthermore, the introduction of specific groups can bolster anti-microbial and antioxidant capabilities, broadening the therapeutic potential of stilbene derivatives. Crucially, these modifications can also refine the neuroprotective effects, offering potential for combating neurodegenerative disorders. Beyond direct bioactivity, these structural changes often lead to enhanced targeting properties, implying that the modified stilbenes may exhibit improved selectivity towards specific cells or tissues, thereby minimizing off-target effects and increasing therapeutic efficacy [98,99].

**Figure 5:**
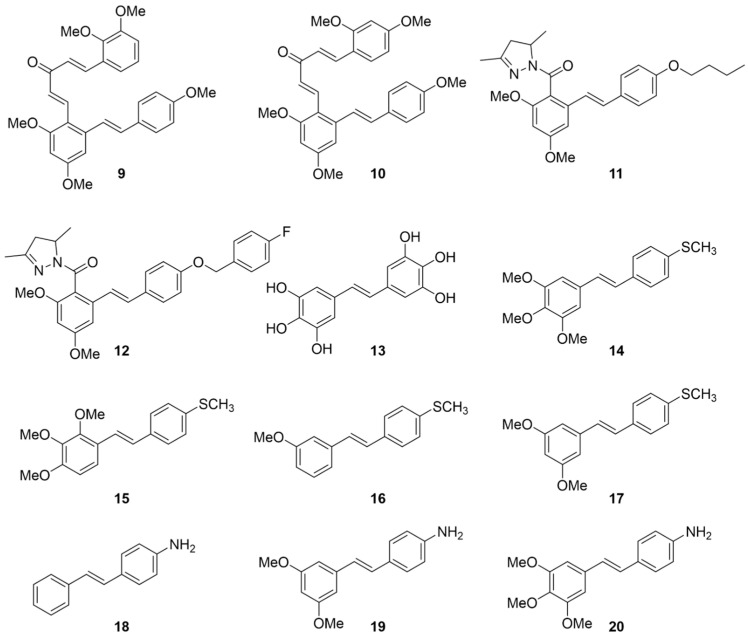
Stilbene analogues through modification around the aromatic ring. Chemical structures were obtained from PubChem database (https://pubchem.ncbi.nlm.nih.gov/, accessed on 1 February 2026) and ChemDraw software (https://revvitysignals.com/products/research/chemdraw, version 20.0, accessed on 1 February 2026).

The initial chemical modification around the aromatic rings focuses on the C-2 and C-4′ positions (Figure 1), yielding novel analogs with improved biological efficacy [100]. In previous research, Wu and co-workers found that a combination of resveratrol with a monocarbonyl moiety at the C-2 position, e.g., compounds **9** and **10** (Figure 5), could lead to an enhanced anti-inflammatory effect [101]. Specifically, compound **9** attenuated the LPS-induced lung damage in a mice model, while compound **10** showed a remarkable inhibition to a wide range of biological activities, such as hepatic inflammation, liver damage, lipid accumulation, and fibrosis associated with a high-fat diet (HFD) in a mouse model [100]. Interestingly, compound **10** significantly reduced the inflammatory markers, including cytokines, COX-2, as well as the expression of palmitic acid-mediated adhesion molecules, e.g., ICAM, VCAM-1, and MCP-1. It is believed that such an anti-inflammatory effect could be mediated through the ERK signaling mechanism [101].

Yang et al. [102] introduced a chalcone and dihydropyrazole fragment into the stilbene scaffold and synthesized a stilbene analog **11** (Figure 5) that exhibited good antioxidant and anti-inflammatory properties. Interestingly, compound **11** reduced the production of ROS in both cultured RAW264.7 and H9C2 cells through promoting the functions of antioxidant enzymes, such as catalase, Nrf2, glutathione peroxidase 1, and SOD1 [102]. In addition, Wei et al. [103] synthesized compound **12** and discovered that the analog could reduce the activities of p-ERK1/2 and β-binding proteins, as well as inflammatory cytokines, i.e., IL-1β, TNF-α, and IL-6. Derivative **12** reduced calcium in the aortic root and notably slowed the progression of atherosclerosis in an animal model [103].

Another interesting strategy to modify the stilbene scaffold is to replace the 4′-hydroxy group at the ring B (Figure 1). The antioxidant and cytotoxic properties of phenolic compounds are intrinsically linked to the characteristics of their hydroxyl (-OH) groups. The presence of these groups is fundamental, as they readily donate a hydrogen atom to neutralize ROS, thereby acting as antioxidants [104,105]. The positioning of these -OH groups on the aromatic ring is equally crucial, influencing their electronic distribution and accessibility. Generally, an increase in the number of -OH groups on the aromatic core amplifies radical scavenging efficiency. More hydroxyl groups mean more sites available for hydrogen atom donation, leading to enhanced protection against oxidative damage [104,105]. However, this increased hydroxylation introduces a duality. While beneficial for antioxidant defense, a higher density of -OH groups can also make the molecule a more attractive substrate for enzymes involved in hepatic metabolism, specifically those in the liver responsible for detoxification and excretion [104,105]. This accelerated metabolism can lead to faster clearance of the compound from the body, potentially reducing its overall efficacy or necessitating higher doses for sustained activity. Therefore, the optimizations of antioxidant and cytotoxic activity involve a delicate balance, considering both the enhanced radical-scavenging power and the potential for increased metabolic breakdown [104,105].

Murias and co-workers conducted research to synthesize and evaluate several hydroxylated stilbene analogues, which indicated that most of these hydroxylated analogues exhibited stronger scavenging activities than resveratrol and a-tocopherol [106]. Specifically, piceatannol and compound **13** were found to be the most potent, demonstrating antiradical activity up to 1250-fold higher than resveratrol. It was concluded that hydroxylated derivatives can be oxidized via a one-electron pathway to form phenoxyl radicals, which can then yield pro-oxidant or alkylating products [106].

In parallel, several lines of evidence have revealed that replacement of the hydroxy group could lead to better biological function and lower cytotoxic effects [107,108]. Mikstacka et al. [108] conducted intensive structure-activity relationship (SAR) studies and produced a series of 4-methylthio analogues as effective inhibitors against the majority of P450 enzymes, such as compounds **14** and **15**. Intriguingly, the potency of these analogs is actually derived from the better binding conformations at the active site of P450 enzymes and direct interaction between the 4-methylthio group and heme iron [108]. In addition, Szaefer and co-worker [109] synthesized 4-methylthio analogues **16** and **17** that demonstrated more effective activities against NF-κB and AP-1, as compared with parental resveratrol. Furthermore, the study revealed the replacement of the 4-methoxy group of ring B with a less electronegative sulfur atom significantly diminished cytotoxicity to HEK 293 cells and also elevated the compound’s potency to stimulate human SIRT1, an NAD^+^-dependent deacetylase regulating cell survival, apoptosis, and stress resistance. Such findings implied that the cytotoxicity of chemical agents could be uncoupled from their capabilities to activate SIRT1 [109].

Amino groups also appear as vital functional groups during chemical modification. Sun and co-workers [110] introduced amino groups into the stilbene scaffold and obtained analogues **18**–**20**. Compound **18**, featuring a *para*-amino group, exhibited a range of biological activities, including the inhibition of nitric oxide synthase, aromatase inhibition, and suppression of TNFα-mediated NF-κB activity [110]. Derivatives **19** and **20** also demonstrated effective QR2 inhibitory activity with a strong binding pose at the active site of the QR2 protein. Sun et al. [105] noted that those analogs with amino groups at ring A generally showed better efficacy than those at ring B, and particularly, amino groups at the *para*-position displayed more effective potency than those at *ortho*- and *meta*-positions [110].

### 4.2. Modification Around Double Bond

**Figure 6:**
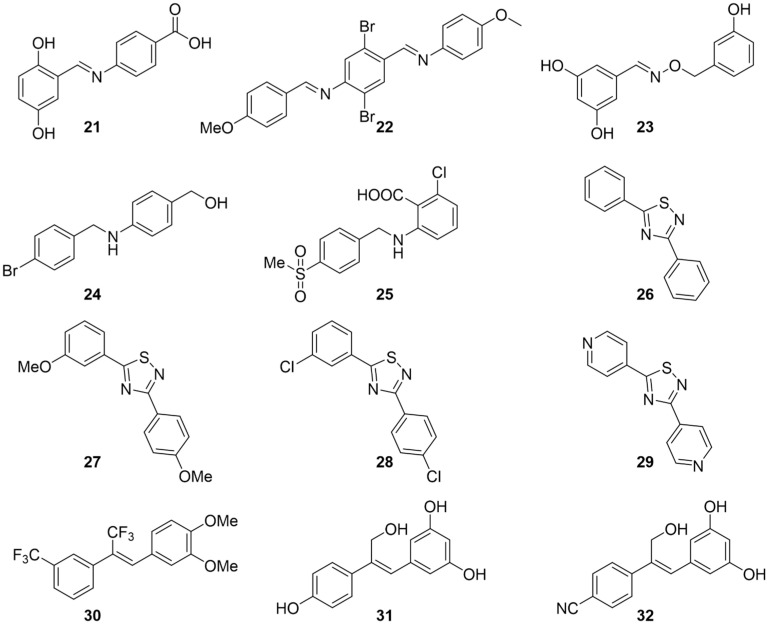
Stilbene analogues through modification around double-bond linker. Chemical structures were obtained from PubChem database (https://pubchem.ncbi.nlm.nih.gov/, accessed on 1 February 2026) and ChemDraw software (https://revvitysignals.com/products/research/chemdraw, version 20.0, accessed on 1 February 2026).

The alkene linker (C=C) in stilbene-type phytochemicals, serving as a connector between two aromatic fragments, has been shown to significantly contribute to the biological functions of stilbenes [111,112]. Several stilbene-type novel analogues are designed by replacing the alkene linker with either a C=N or N=N group using a bioisosteric substitution approach (Figure 6). Additionally, rigidity of carbon-carbon double bonds can also be replaced with more flexible chemical groups, including amides, amines, and simple carbon-carbon single bonds, leading to flexible binding posts towards targeted proteins and improved biological effectiveness in stilbene derivatives [111,112].

For instance, Iacopetta et al. [113] synthesized imino **21** (Figure 6) that exhibited robust anticancer effects in both MCF-7 and SkBr-3 cell lines, and the IC_50_ values were 12 μM and 15 μM, respectively [113]. Furthermore, compound **21** showed better bioavailability than the parent resveratrol under gastric as well as small intestinal conditions, with a 35% increase compared with resveratrol [113]. Subsequently, compound **22** was synthesized by Gonzalez et al. [114] and displayed antimicrobial effectiveness against *Listeria monocytogenes* with an EC_50_ value of 10.07 μg/mL, while analogue **23** showed promising antioxidant activity in cultured HUVECs. Wu et al. [115] synthesized a series of derivatives with an amine group as a linker, of which **24** and **25** significantly reduced nitric oxide (NO) production at a concentration of 10 μM, exhibiting concentration-dependent inhibition [115]. Furthermore, both compounds inhibited COX-2 and triggered cytotoxicity in MCF-7 cancer cells [115].

Mayhoub et al. [116] conducted a cyclizing strategy to introduce a heterocycle and replace the double linker and obtained a number of thiadiazole stilbene molecules. Through substituting the stilbene ethylenic bridge of resveratrol with a 1,2,4-thiadiazole heterocycle, compound **26** was designed and yielded, which further resulted in enhanced inhibition to QR1 and NF-κB, as well as aromatase [116]. Further modifications, such as adding two methoxy groups or halogen atoms to the aromatic rings of compound **26**, led to the synthesis of **27**, **28**, and **29**, which improved both efficacy and selectivity to QR1, NF-κB, and aromatase [116]. Interestingly, Mayhoub et al. [116] summarized that 4,4′-disubstituted analogues in this series generally displayed stronger effectiveness than any other analogues.

In another study, an olefin group was introduced into the stilbene scaffold and yielded a group of olefin-substituted analogs **30**–**32** [117,118]. Compound **29** appeared as the most potent inhibitor against QR2 protein, with an IC_50_ value of 0.18 µM [117]. Other molecules **31**–**32** containing electron-donating groups (such as OH and OMe) effectively inhibited QR2 with increased affinity as compared to resveratrol, positioning them as potential leads for the subsequent pharmacological development of anticancer treatments [118].

### 4.3. Dimerization of Stilbene Analogues

Stilbene polymers have attracted significant attention owing to their wide range of biological performance. For example, resveratrol polymers primarily exist as oligomers, with some oligomeric forms demonstrating greater activity than resveratrol itself [119,120]. This evidently suggests that the stilbene scaffold itself appears as a potent fragment and, therefore, a novel analog with dimerization of the stilbene moiety could potentially enhance the biological activities of stilbenes [119,120].

**Figure 7:**
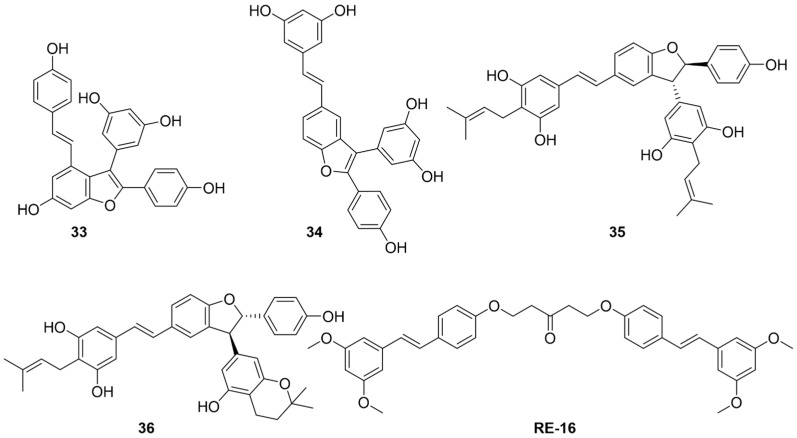
Stilbene analogues through dimerization of stilbene scaffold. Chemical structures were obtained from PubChem database (https://pubchem.ncbi.nlm.nih.gov/, accessed on 1 February 2026) and ChemDraw software (https://revvitysignals.com/products/research/chemdraw, version 20.0, accessed on 1 February 2026).

Fan and co-workers first discovered a resveratrol dimer, i.e., compound **33** (Figure 7), showed remarkable antioxidant and anti-inflammatory potency [121]. Their study revealed that compound **33** was able to reduce inflammatory cytokines, including IL-6, IL-17A, and TNF-α in lung tissue. Moreover, compound **33** notably decreased the levels of p-NF-κB, NF-κB, and p-Syk in cell assays as well as animal models [121].

In parallel, Mattio et al. [122] synthesized a series of dimers, with the dimer **34** demonstrating considerable antimicrobial efficacy against gram-positive bacteria. Specifically, compound **34** exhibited significant anti-bacterial potency against *Listeria monocytogenes* at a concentration of 2 μg/mL. On the other hand, compound **34**, at a concentration of 100 μg/mL, could induce severe damage to cell membranes, including damage of the membrane, disruption of membrane integrity, and significant morphological changes in bacteria [122]. Tang et al. [123] reported a series of resveratrol dimers, such as derivatives **35** and **36**, both of which displayed effective potency to human monoamine oxidase B and contained robust antioxidant activities. Besides, the dimerizing analogues exhibited promising bioavailability and low toxicity in cell studies [123]. Another potential dimer, named RE-16 with an alkane linker, was designed and synthesized by our team. Specifically, RE-16 displayed more robust anti-VEGF efficacy in both in vitro and cell models than the hit molecule and therefore appeared as a potential agent against VEGF-mediated disease for the following pharmacological development [124].

## 5. Discussion

Stilbene-type compounds are a diverse group of plant secondary metabolites that play significant roles in both plant physiology and human health [125]. These compounds, emerging primarily from various plant sources, belong to the broader category of polyphenols that are known for their complex structures and extensive range of biological activities. Notably, stilbenes are found in multiple food sources, including grapes, peanuts, and several medicinal plants such as *Hopea chinensis* and *Polygonum cuspidatum* [125,126]. Their presence in these plants not only highlights their ecological significance but also underscores their potential medicinal benefits, drawing interest in further investigations into their applications in the fields of medicine and nutrition.

The biological activities of stilbene-type compounds are various and well-documented [127,128]. One of the most researched stilbene derivatives, resveratrol, is renowned for its antioxidant, anti-inflammatory, and anti-cancer properties, owing to its ability to modulate various signaling pathways and influence metabolic processes within cells [127]. In recent years, studies have emphasized the potential of stilbenes to combat chronic diseases, such as cancer and neurodegenerative disorders, which have made them reliable hit molecules for the following pharmacological development [128].

However, the natural occurrence of stilbene compounds presents certain limitations in their biological effectiveness, particularly in terms of bioavailability and solubility [129,130]. This has prompted research into the chemical modification of stilbene scaffolds to overcome these challenges, as structural modifications around the core stilbene skeleton can significantly enhance the desired properties of these compounds [129]. The strategic modification of the stilbene moiety, especially at its aromatic rings, is a primary method in enhancing its bioactivities. This involves introducing functional groups at the C-2 and replacing the 4’-hydroxy group at the ring B. These modifications aim to create synergistic interactions with the stilbene core, potentially leading to significant improvements in therapeutic applications. Enhanced anti-inflammatory and anticancer properties are frequently observed, indicating that these alterations can modulate the cellular signaling pathways involved in diseases. The ethylene bridge between the two aromatic rings is a crucial composition of stilbenes. Through replacing the ethylene bridge with more flexible chemical groups, it was observed that the compounds generally contained flexible binding posts towards targeted proteins and improved biological effectiveness. Last but not least, the stilbene fragment itself appears as a potent motif, and therefore a novel analog with dimerization of the stilbene moiety has been found to promote the biological activities of stilbenes. Studies on these modifications not only reveal the biological activities of the compounds but also outline the future perspectives for subsequent structural modification around stilbenes with an aim to further improve their interactions with various cellular targets and clinical efficacy [129,130].

Current SAR studies of stilbenes face significant limitations, primarily due to their poor aqueous solubility, low bioavailability, and highly variable pharmacokinetic profiles [131,132]. The reliance on in vitro data, where assays often fail to accurately predict clinical applications, presents a substantial hurdle for advancing stilbene-based drug design. Key challenges include the translation gap between in vitro findings and in vivo efficacy, as results from isolated enzymes or cell cultures do not always correlate with human plasma concentrations due to complex Absorption, Distribution, Metabolism, and Excretion (ADME) profiles [131,132]. Furthermore, stilbenes, particularly resveratrol, are rapidly metabolized in the liver and gut through glucuronidation and sulfation, rendering them inactive before they can exert pharmacological effects [133,134]. Finding universal rules for substitution patterns is also elusive, as the impact of functional groups like hydroxyl and methoxy on binding affinity or cytotoxicity is highly specific to the targeted disease. Lastly, the inherent instability of stilbenes, which undergo *cis-trans* isomerization upon UV absorption, leads to different binding efficacies between isomers, thus limiting the predictive power of computational models and drug screenings [133,134]. These multifaceted challenges necessitate overcoming specific structural and metabolic hurdles to effectively develop stilbene-based therapeutics.

## 6. Conclusions

In conclusion, the ongoing study of stilbene-type compounds emphasizes their value not only in traditional medicine but also as candidates for modern drug discovery. As research progresses, these compounds serve as promising chemical agents for new therapeutic approaches to combat various diseases, ultimately contributing to improved health outcomes and expanded applications in nutraceuticals and pharmaceuticals. The synergistic relationship between their natural origins and the potential for innovative chemical modification underscores the importance of continued exploration in this field. Current stilbene SAR studies are hampered by poor solubility, bioavailability, and rapid metabolism, leading to a significant gap between in vitro predictions and in vivo efficacy. Overcoming these limitations, including isomer instability and variable pharmacokinetic profiles, is crucial for developing effective stilbene-based therapeutics.

## Figures and Tables

**Table 1:** Biological activities of stilbene analogues.

Activities	Chemicals and References	Experimental Models	Potency
Antioxidant	Resveratrol [44,45]	B16 melanoma cell model	IC_50_ = 63.2 µM
	Piceatannol [44]	B16 melanoma cell model	IC_50_ = 1.53 µM
	Compound **4** [48]	In vitro DPPH assay	IC_50_ > 200 µM
	Compound **5** [48]	In vitro DPPH assay	IC_50_ = 69.0 µM
	Compound **6** [48]	In vitro DPPH assay	IC_50_ = 126.6 µM
	Compound **7** [48]	In vitro DPPH assay	IC_50_ > 200 µM
	Compound **8** [48]	In vitro DPPH assay	IC_50_ = 43.5 µM
Anti-inflammatory	Resveratrol [49]	LPS-stimulatedTHP-1 cells	IC_50_ = 19 µM
	Polydatin [50]	TNF-α and IL-1β in renal tissue	Showed effect at dosages of 50.0 and 25.0 mg/kg/day
	Viniferin [51]	LPS-stimulated murine macrophages	Showed effect at dosages of 1 and 10 µM
	Piceatannol [51,52]	LPS-stimulated murine macrophages	Showed effects at dosages of 1 and 10 µM
	Rhapontigenin [53]	Histamine release from rat peritoneal exudate cell model	IC_50_ = 0.25 µM
Anti-cancer	Resveratrol [54,55,56]	HT29 colon cancer cells from nude mice	Showed effect at dosage of 80 mg/kg/day
	Pterostilbene [57]	MCF7 breast cancer cell lines	Showed effect at dosage of 5 and 10 µM
	Piceatannol [58,59]	Antiproliferative effect in MTT assay	IC_50_ = 31.6 µM
	Prenylated hydroxystilbene [58]	Antiproliferative effect in MTT assay	IC_50_ = 21.0 µM
Cardiovascular disease	Resveratrol [60,61]	randomized human clinical trials	Showed effect at dosage of 8 mg/day
	Silymarin [62,63]	randomized human clinical trials	Showed effect at dosage of 105 mg twice a day for 3 months
	Pterostilbene [64]	Randomized and placebo-controlled trial	Showed effect at dosage of 125 mg twice a day
Neuroprotective effect	Resveratrol [65,66]	MPP+-induced MES23.5 DA cells	Showed effect at dosage of 25 µM
	Pterostilbene [67,68]	hyperglycemia-induced SHSY5Y cell model	Showed effect at concentrations 2.5–10 µM
	Oxyresveratrol [69,70]	Rat MCAO model	Showed effect at dosage 2–30 mg/kg/day
Obesity	ε-viniferin [71,72]	Diet-induced obesity in C57BL/6J mice	IC_50_ = 33.5 ± 9.7 µM
	Pterostilbene [73,74]	Diet-induced obesity in C57BL/6J mice	Showed effect at dosage 15–30 mg/kg/day
Diabetes	Resveratrol [75]	Male C57BL/6J mice model	Showed effect at dosage 8–16 mg/kg/day
	Pterostilbene [76,77]	Male Wistar rat model	Showed effect at dosage 10–40 mg/kg/day
Anti-melanogenetic	Resveratrol [78]	UVB-induced brownish guinea pig model	Showed effect at dosage 1% solution (200 μL)/day
	Piceatannol [79]	B16F10 mouse melanoma cell model	Showed effect at dosage 5–50 µM

## Data Availability

All data is available from corresponding author upon reasonable request.
